# Oral Microbiota of the Snake *Bothrops lanceolatus* in Martinique

**DOI:** 10.3390/ijerph15102122

**Published:** 2018-09-27

**Authors:** Dabor Résière, Claude Olive, Hatem Kallel, André Cabié, Rémi Névière, Bruno Mégarbane, José María Gutiérrez, Hossein Mehdaoui

**Affiliations:** 1 Intensive Care Unit, University Hospital of Martinique, Fort-de-France, 97200 Martinique, France; Remi.NEVIERE@chu-martinique.fr (R.N.); Hossein.MEHDAOUI@chu-martinique.fr (H.M.); 2 Department of Microbiology, University Hospital of Martinique, 97200 Martinique, France; Claude.OLIVE@chu-martinique.fr; 3 Intensive Care Unit, Rosemond André General Hospital, Cayenne, 97300 French Guiana, France; hatem.kallel@ch-cayenne.fr; 4 Department of Infectious Diseases, University Hospital of Martinique, 97200 Martinique, France; Andre.CABIE@chu-martinique.fr; 5 Department of Medical and Toxicological Critical Care, Lariboisière Hospital, Paris-Diderot University, INSERM UMRS-1144, 75010 Paris, France; bruno.megarbane@lrb.aphp.fr; 6 Instituto Clodomiro Picado, Facultad de Microbiología, Universidad de Costa Rica, 11501 San José, Costa Rica; jose.gutierrez@ucr.ac.cr

**Keywords:** *Bothrops lanceolatus*, envenomation, snakebite, bacteria, infection, antibiotic susceptibility

## Abstract

In Martinique, *Bothrops lanceolatus* snakebite, although relatively uncommon (~30 cases/year), may result in serious complications such as systemic thrombosis and local infections. Infections have been hypothesized to be related to bacteria present in the snake’s oral cavity. In this investigation, we isolated, identified, and studied the susceptibility to beta-lactams of bacteria sampled from the oral cavity of twenty-six *B. lanceolatus* specimens collected from various areas in Martinique. Microbiota from *B. lanceolatus* oral cavity was polymicrobial. Isolated bacteria belonged to fifteen different taxa; the most frequent being *Aeromonas hydrophyla* (present in 50% of the samples), *Morganella morganii*, *Klebsiella pneumoniae*, *Bacillus* spp., and *Enterococcus* spp. Analysis of antibiotic susceptibility revealed that 66.7% of the isolated bacteria were resistant to amoxicillin/clavulanate. In contrast, the majority of isolated bacteria were susceptible to the third-generation cephalosporins (i.e., 73.3% with cefotaxime and 80.0% with ceftazidime). Microbiota from *B. lanceolatus* oral cavity is polymicrobial with bacteria mostly susceptible to third-generation cephalosporins but rarely to amoxicillin/clavulanate. In conclusion, our findings clearly support that first-line antibiotic therapy in the *B. lanceolatus*-bitten patients, when there is evidence of infection, should include a third-generation cephalosporin rather than amoxicillin/clavulanate.

## 1. Introduction

**Figure 1:**
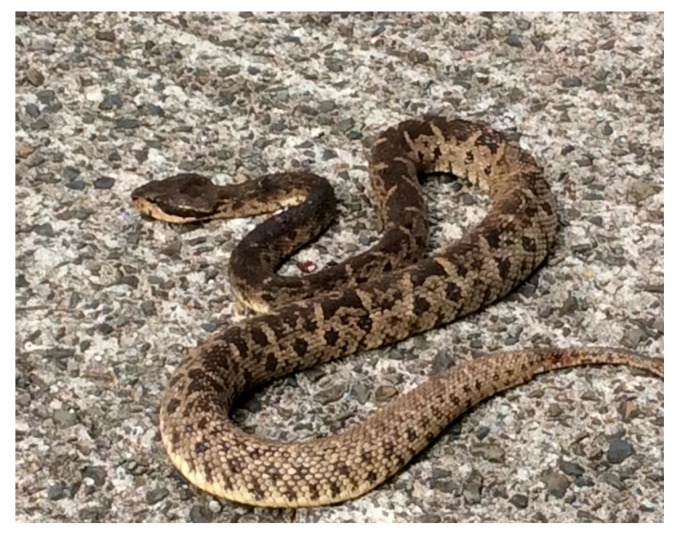
*Bothrops lanceolatus* from Martinique, known as trigonocéphale or fer-de-lance is one the most dangerous venomous snakes in the Caribbean. (Photo courtesy of Mr. Michel Tanasi, a member of the snake working group).

Snakebite envenomation is a relatively uncommon but serious medical emergency in Martinique, an overseas region of France with a population of 386,000 inhabitants, located in the Lesser Antilles of the Eastern Caribbean. Approximately thirty cases are declared every year. Envenomation is due to *Bothrops lanceolatus* (Order: Squamata; suborder: Serpentes; family: Viperidae; subfamily: Crotalinae; Figure 1), the only venomous snake in this Caribbean French territory and a snake not found elsewhere in the world [1]. This envenoming is known to be responsible for a high rate of multiple systemic thrombotic events as well as for possible local infectious complications. 

As suggested by previous observations at the bedside [2,3], bacterial infection from snakebites in Martinique occurs in about one third of the envenomed cases, mainly in the most severely envenomed patients (grade II or III). Interestingly, local infection was hypothesized to be caused by the oral and fang microbiota of *B. lanceolatus*.

The oral microbiota of snakes comprises a wide range of aerobic and anaerobic microorganisms, including *Enterobacteriaceae* (*Morganella* spp. and *Escherichia coli*), *Streptococcus* spp., *Aeromonas* spp., *Staphylococcus aureus*, and *Clostridium* spp., as reported in several observational studies from snakebites worldwide [4,5,6,7,8,9]. However, the predominant microorganisms change according to the geographic region and environmental conditions. Additionally, bacteria susceptibility to beta-lactams, the most frequently used antibiotics to treat snake-envenomed patients, varies with possible resistant phenotypes, especially since the emergence of multidrug-resistant bacteria in the environment during the last years. 

Therefore, we designed an experimental study aiming to identify the main bacterial microbiota from *B. lanceolatus* oral cavity. Then, based on the nature and susceptibility of the isolated bacteria to beta-lactams, our objective was to propose the most adequate preemptive antibiotic therapy that should be administered in the suspicion of infection in *B. lanceolatus*-bitten patients in Martinique.

## 2. Materials and Methods

### 2.1. Experimental Design 

This experimental study was conducted at the Microbiology Laboratory of the University Hospital of Martinique. During eighteen months, all *B. lanceolatus* snakes captured by personnel of the National Office of Forests in Martinique were studied. Snakes were grouped according to their geographical origin, i.e., wet (forest and near water areas) versus dry regions (peri-urban areas). The snake’s oral cavity was opened and sampled from the vicinity of the fangs using sterile cotton swabs. Samples were subjected to Gram staining and examined for bacterial growth. They were plated on non-selective blood agar and chocolate agar and cultured at 37 °C for 2–7 days and the color and shape of the colonies were observed. Species identification was performed with API-20E and API-20NE systems (BioMérieux, Marcy L’Etoile, France). Antimicrobial susceptibilities of all isolates to beta-lactams were determined by the disk diffusion methods based on the definitions of the Antibiogram Committee of the French Microbiology Society [10]. The inhibition zone diameters of each drug for each isolate were determined after overnight incubation at 35.8 °C in ambient air. The interpretive criteria of the inhibition zone and minimum inhibitory concentrations were in accordance with those of the Antibiogram Committee of the French Society of Microbiology.

### 2.2. Statistical Analysis

Data are expressed as absolute values and percentages. Comparisons between the subgroups were performed using Chi-2 tests. *p*-Values less than 0.05 were considered to be statistically significant.

## 3. Results

During the study period, twenty-six specimens of *B. lanceolatus* were captured and their oral cavity sampled for bacteriological culture. Twenty snakes were captured from the “wet” zones versus six from the “dry” zones. All samples obtained from the snake mouths tested positive for bacterial growth. 

In 20 cases (76.9%), the sample was polymicrobial. The most frequently isolated bacteria were *Aeromonas hydrophila*, *Morganella morganii*, and *Klebsiella pneumonia* (Table 1). *A. hydrophila* was isolated in 50% of the samples (13 cases). In four cases (15.4%), pure cultures were obtained or the bacterium was quantitatively predominant. *A. hydrophila* was isolated in 60.0% of cases from snakes captured from a “wet” zone compared to 16.7% from snakes captured from a “dry” zone (*p* = 0.06). 

In 66.7% of the cases, the isolated microorganisms were resistant to amoxicillin/clavulanate, while bacteria isolated were more frequently susceptible to the third-generation cephalosporins including cefotaxime (73.3%) and ceftazidime (80.0%).

## 4. Discussion

In this study, the most frequently isolated microorganisms from *B. lanceolatus* oral cavity in Martinique were *A. hydrophila*, followed by other members of the *Enterobacteriaceae* family. The cultures were polymicrobial and bacteria were susceptible to third-generation cephalosporins in most cases, coinciding with the majority of studies that focused on other snakes from different regions of the Americas [8,10,11,12]. 

*B. lanceolatus* is endemic to Martinique in the French West Indies. Its venom can cause severe local damage, ranging from blistering and tissue necrosis to secondary cellulitis and abscess in severe cases [1]. As previously shown [2,3,4,5,6,7], the oral microbiota of snakes is known to contain a wide range of microorganisms. Conformingly, *B. lanceolatus* oral flora is polymicrobial, combining Gram-positive, Gram-negative bacteria were isolated, with varying proportions across the Martinique sub-regions. 

*A. hydrophila* was the most commonly isolated bacterial species, especially when the snake was captured from a “wet” zone. This bacterium is known to cause tissue damage and necrotizing fasciitis [13,14,15], raising the hypothesis that the tissue damage observed after *B. lanceolatus* bite may result from the tissue-damaging properties of the venom combined with the necrosis potency of *A. hydrophila*. It is obvious that tissue damage induced by the venom is responsible for perfusion abnormalities in the zone of the bite. This is due to microvascular damage and thrombosis induced by the venom itself, in addition to microcirculatory compression due to local edema and compartment syndrome in some cases. In these conditions, tissue damage and ischemia, with an associated decrease in the local innate immunity, is an excellent medium for the growth of bacteria ejected from the snake’s oral cavity at the moment of the bite, as previously demonstrated for *Staphylococcus aureus* in an experimental model [16]. 

In the case of a moderate to severe snakebite, most authors and guidelines recommend the use of antibiotics to reduce complications by preventing secondary infection. However, the 2010 World Health Organization statements advised against the use of preemptive antibiotics in snake bites except in certain circumstances [17]. Our study clearly showed that envenomings by *B. lanceolatus* species have a high incidence of bite abscesses and, thus, represent an ideal candidate to use antibiotics, if they are given when there is the evidence of infection soon enough after the incident and when appropriate antibiotics are employed.

The most recommended antibiotic for snakebite treatment is amoxicillin/clavulanate, albeit without strong evidence supporting this recommendation. The Infectious Diseases Society of America (IDSA) guidelines for the diagnosis and management of skin and soft-tissue infections recommended amoxicillin/clavulanate as the preemptive antibiotic of choice to reduce complications by preventing secondary infection from animal bites other than snakes [18]. However, preemptive amoxicillin/clavulanate was reported to be ineffective in preventing secondary infections from *Bothrops* snakebites in the Western Brazilian Amazon region [19]. Similarly, in another study based on a 10-year experience in a northern Taiwan medical center, amoxicillin/clavulanate alone appeared non-convenient for the empirical or definitive treatment of soft tissue infections after snakebite [20]. In this setting, the authors advised to use the combination of amoxicillin/clavulanate with ciprofloxacin or to choose parenteral piperacillin/tazobactam. 

Our findings were consistent with these two studies [19,20], since we showed that amoxicillin/clavulanate was not effective against 66.7% of the isolated bacteria. By contrast, we found that the most appropriate first-line antibiotics were the third-generation cephalosporins that should be preferred in *B. lanceolatus*-bitten patients with signs of local infection. However, since the systematic prophylactic antibiotic use in snakebite patients remains a matter of debate [21], the use of antibiotics in *B. lanceolatus* cases should be restricted to cases where there is evidence of infection. 

## 5. Conclusions

Microbiota from *B. lanceolatus* oral cavity is polymicrobial with bacteria mostly susceptible to third-generation cephalosporins and more rarely to amoxicillin/clavulanate. In the absence of definitive international guidelines for the management of snakebite-associated infections, our findings support that the first-line antibiotic therapy should include a third-generation cephalosporin rather than amoxicillin/clavulanate in patients suffering infections as a consequence of *B. lanceolatus* snakebites in Martinique.

## Figures and Tables

**Table 1:** Bacteria isolated from the oral cavity of *Bothrops lanceolatus* in Martinique and their susceptibility to beta-lactams.

Microorganism	*N*	AMX	AMX-Clav	CTX	CAZ	% of Bacteria (*N* = 46)	% of Specimens (*N* = 26)
*Aeromonas hydrophila*	13	R	R	S	S	28.3	50
*Morganella morganii*	7	R	R	S	S	15.2	26.9
*Klebsiella pneumoniae*	5	R	S	S	S	10.9	19.2
*Bacillus* spp.	4	R	R	R	R	8.7	15.4
*Enterococcus* spp.	3	S	S	R	R	6.5	11.5
*Proteus mirabilis*	2	S	S	S	S	4.3	7.7
*Serratia marcescens*	2	R	R	S	S	4.3	7.7
*Shewanella putrefaciens*	2	R	R	S	S	4.3	7.7
*Clostridium bifermentans*	2	R	R	S	S	4.3	7.7
*Proteus penneri*	1	S	S	S	S	2.2	3.8
*Proteus vulgaris*	1	S	S	S	S	2.2	3.8
*Enterobacter cloacae*	1	R	R	S	S	2.2	3.8
*Citrobacter freundii*	1	R	R	S	S	2.2	3.8
*Chryseomonas violaceum*	1	R	R	R	R	2.2	3.8
*Pseudomonas pickettii*	1	R	R	R	S	2.2	3.8
Total	46	-	-	-	-	100	100
% susceptible	-	26.7	33.3	73.3	80.0	-	-

S, susceptible; R, resistant; AMX, amoxicillin; AMX-Clav, amoxicillin/clavulanate; CTX, cefotaxime; CAZ, ceftazidime.
